# Dual-Band Dual-Mode Substrate Integrated Waveguide Filters with Independently Reconfigurable TE_101_ Resonant Mode

**DOI:** 10.1038/srep31922

**Published:** 2016-08-26

**Authors:** Yongle Wu, Yuqing Chen, Lingxiao Jiao, Yuanan Liu, Zabih Ghassemlooy

**Affiliations:** 1 Beijing Key Laboratory of Work Safety Intelligent Monitoring, School of Electronic Engineering, Beijing University of Posts and Telecommunications, P.O. Box. 282, 100876, Beijing, China; 2 Optical Communications Research Group, NCRLab, Faculty of Engineering and Environment, Northumbria University, Newcastle upon Tyne, NE1 8ST, U.K

## Abstract

A novel perturbation approach using additional metalized via-holes for implementation of the dual-band or wide-band dual-mode substrate integrated waveguide (SIW) filters is proposed in this paper. The independent perturbation on the first resonant mode TE_101_ can be constructed by applying the proposed perturbation approach, whereas the second resonant mode TE_102_ is not affected. Thus, new kinds of dual-band or wide-band dual-mode SIW filters with a fixed or an independently reconfigurable low-frequency band have been directly achieved. In order to experimentally verify the proposed design method, four two-cavity dual-band SIW filters, which have different numbers of perturbation via-holes in each cavity, and a two-cavity dual-band SIW filter, which includes four via-holes and eight reconfigurable states in each cavity, are designed and experimentally assessed. The measured results indicate that the available frequency-ratio range from 1 to 1.3 can be realized by using four two-cavity dual-band SIW filters. The center frequency of the first band can be tuned from 4.61 GHz to 5.24 GHz, whereas the center frequency of the second one is fixed at around 6.18 GHz for the two-cavity dual-band SIW filter with four reconfigurable states via-holes. All the simulated and measured results show an acceptable agreement with the predicted data.

Concurrent multi-band or real-time tunable band radio-frequency/microwave/terahertz transceivers provide effective and productive solutions to overcome the efficient-utilization problem in spectrum congested wireless communications in the real world. As a key building block of modern wireless transceiver systems, concurrent multi-band or real-time tunable band filters require multi-function integrated features to satisfy the increasing demands for the spectrum in future emerging applications in telecommunication systems and Internet of Things. Recently, a number of multi-band and tunable/reconfigurable filters have been reported in the literatures including coplanar waveguide filters^1^, microstrip filters^2^, and substrate integrated waveguide (SIW) filters^3^. Due to the advantages such as easy fabrication, simple integration with active devices, compact size, low cost, mass-production, small loss, higher quality factor, high-skirt filtering selectivity, complete shielding, and enhanced power capability of the SIW technology, novel SIW-based multi-band^3^,^4^,^5^ or tunable/reconfigurable filters^6^,^7^,^8^,^9^,^10^,^11^ have received particular attention. In general, compared with SIW-based multi-band filters, which possess low losses, mass production, high quality factor, and high power capacity, SIW-based tunable/reconfigurable filters, which use a single dielectric to merge waveguide cavities with planar structures, can selectively cover multiple frequency bands while maintaining lower crosstalk sensitivity.

There are only two categories of SIW-based tunable/reconfigurable filters: 1) the electrically tunable filters^6^,^7^,^8^, and 2) the mechanically tunable ones^9^,^10^,^11^,^12^. By applying controllable DC voltages at each varactor on complementary split-ring resonators, the pass-band tuning performance can be achieved for these SIW-based electrically tunable filters^6^,^7^,^8^. For mechanically tunable filters^9^,^10^,^11^,^12^, the chosen switching elements directly connect the additional metallized via hole with the top metalized layer on the cavity, which can be used as trimming technique and to develop tunable filters. Their operating-frequency adjustability is achieved by discretely controlling the switch states. However, all the reported methods^6^,^7^,^8^,^9^,^10^,^11^,^12^ for SIW-based tunable/reconfigurable filters lead to complex circuit structures and high cost multi-layer structures. Furthermore, only a single pass-band feature can be realized by using these methods.

In order to construct a new single-layer dual-band or wide-band dual-mode SIW filter, a novel perturbation approach, which uses additional metalized via-holes, is proposed in this paper. Unlike the previous methods in the papers^6^,^7^,^8^,^9^, where only the effect of additional metalized holes on one mode (the TE_101_ mode) is studied, the effects of additional metalized holes on two modes (the TE_101_ and TE_102_ modes) are considered in this paper and the two modes are utilized for generating dual pass bands. Moreover, the length of slots in dual-band SIW filter^5^ only affects the upper passband but does not influence the lower passband. In contrast, additional metalized holes can only influence the first resonant mode TE_101_, but cannot change the second resonant mode TE_102_ in the proposed SIW filters. The use of the proposed perturbation holes at the center of each cavity significantly increases the flexibility of developing dual-band or wide-band tunable band SIW filters. Based on the outlined description of the operating principle and mechanism, two kinds of dual-band SIW filters, i.e., four two-cavity SIW filters with fixed dual bands and a two-cavity reconfigurable dual-band SIW filter, have been designed, fabricated, and experimentally evaluated. The measured results show that four two-cavity dual-band SIW filters with a frequency-ratio ranging from 1 (wide-band case) to 1.3 (dual narrow band case with the largest separation) can be realized. On the other hand, the center frequency of the first band can be tuned from 4.61 GHz to 5.24 GHz, but the center frequency of the second band will be unaltered and fixed at around 6.18 GHz for the two-cavity dual-band SIW filter with four reconfigurable via-holes. All the simulated and measured results are in good agreement with the predicted data, thus verifying the proposed concept and the effectiveness of the design method. To sum up, the proposed perturbation approach by using the additional metalized via-holes in dual-band or wide-band even tunable band SIW filters offers a number of advantages including: 1) high selectivity of dual band or wide band, 2) low-cost fabrication using the printed circuit board technology, 3) easy integration with the single-layer circuits and systems, 4) convenient implementation of the tunable or reconfigurable band, and 5) enhanced power capability and decreased radiation loss due to complete shielding with standard metalized via-holes.

## Method

### Theoretical design and the principle of operation

**Figure 1:**
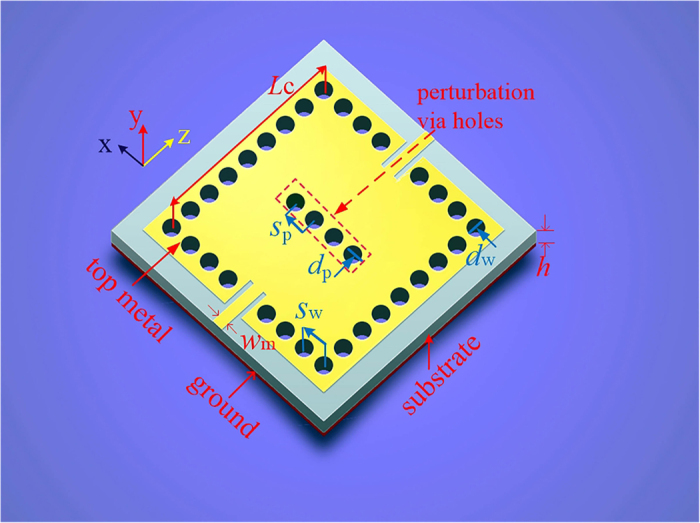
A single-cavity SIW filter with four perturbation via holes. This filter is excited by two 50 Ω microstrip feeding lines. The ground is constructed completely by a metal and the used substrate is Rogers 4350B with the thickness *h* of 0.762 mm. *L*_c_ is the length (width) of the single square cavity, *w*_m_ is the width of the microstrip feeding lines, *h* is the thickness of the chosen substrate, *d*_w_ is the diameter of wall via holes, *d*_p_ is the diameter of perturbation via holes, and *s*_w_ (*s*_p_) is the distance between the center positions of two adjacent wall via holes (perturbation via holes). *L*_c_ = 28.4 mm, *d*_w_ = *d*_p_ = 0.6 mm, *s*_w_ = 1.2 mm, *s*_p_ = 1 mm, and *w*_m_ = 1.72 mm.

Fig. 1 illustrates the geometry of the proposed single-cavity SIW filter with four perturbation via-holes on a single square cavity. The physical parameters’ definition and detailed dimensions are depicted in Fig. 1. The initial sizes of this square cavity in Fig. 1 can be calculated by the following equation^4^:


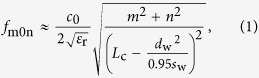
where *f*_m0n_ is the resonant frequency of the TE_m0n_ mode, *c*_0_ is the light velocity in a vacuum, *ε*_r_ is the relative dielectric constant of the chosen substrate, *L*_c_ is the length (width) of the single square cavity, *d*_w_ is the diameter of wall via-holes, *s*_w_ is the diameter of wall via-holes, and *m* and *n* are the indices of the modes. In addition, to avoid dispersion loss of typical SIW structures, the parameters of the wall via-holes are required to meet the following condition in practical applications^3^:


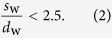
Therefore, when the desired resonant frequencies *f*_m0n_ of the resonant modes TE_m0n_ and the values of necessary parameters including *d*_w_, *s*_w_, *m* and *n* are known, the initial sizes of the square cavity can be readily determined using equations (1) and (2).

**Figure 2:**
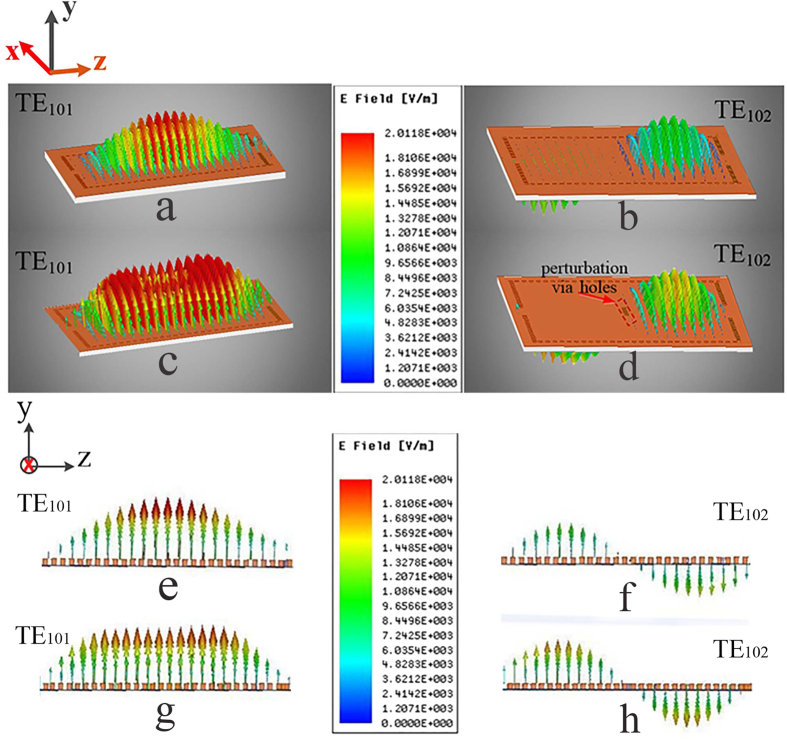
Three-dimensional (3D) and planar comparison of two-mode electric-field vector distributions in a single cavity given in Fig. 1. (a,b) 3D electric-field vector distributions of the TE_101_ mode and the TE_102_ mode without any perturbation via holes, respectively. (**c,d**) 3D electric-field vector distributions of the TE_101_ mode and the TE_102_ mode with four perturbation via-holes in the middle place of this cavity (perpendicular to the *z*-axis, and parallel to the *x*-axis), respectively. (**e–h**) The spatial variations of the YOZ-Plane planar electric field vector distributions, which correspond to (**a–d**).

In this proposed dual-band or wide-band dual-mode SIW filter, the first and the second passbands are generated by the TE_101_ and TE_102_ modes, respectively. The three-dimensional (3D) and planar two-mode electric-field vector distribution in a single cavity are illustrated in Fig. 2. Adding perturbation via-holes at the center of the cavity (i.e., perpendicular to the *z*-axis, and parallel to the *x*-axis) cannot affect the electric-field vector distributions of the TE_102_ mode, because the center of the cavity is equivalent to a short-circuited point for the TE_102_ mode. In contrast, the electric-field vector distribution of the TE_101_ mode is influenced obviously by these additional via-holes. The significance of the flat distribution of the TE_101_ mode with perturbation via-holes shown in Fig. 2(g) is decreasing the effective equivalent physical width of this cavity. This is the core principle of the proposed dual-band dual-mode SIW filters.

## Results

### The fixed dual-band or wide-band dual-mode SIW filters

**Figure 3:**
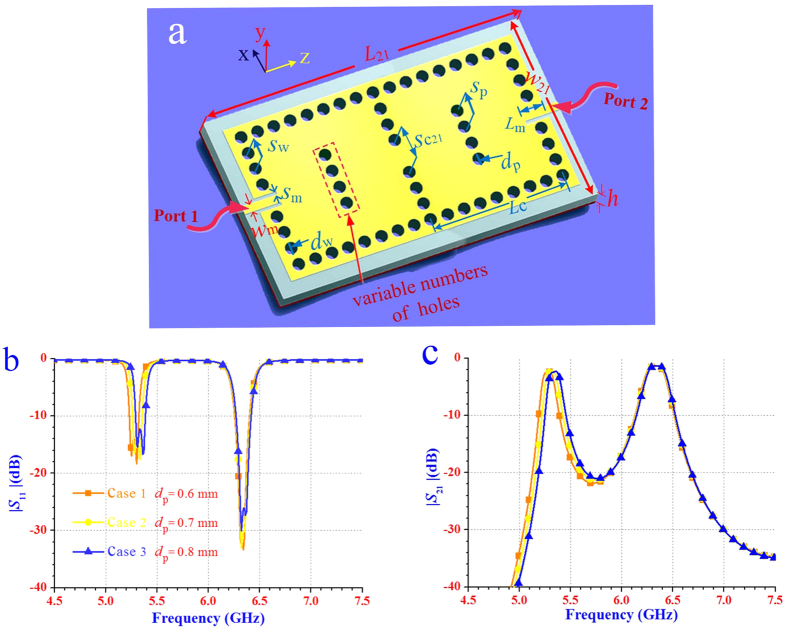
The two-cavity dual-band SIW filter with four perturbation via-holes for sensitivity analysis. (**a**) The SIW filter composed of two cavities with the desired dimensions: *d*_w_ = 0.6 mm, *L*_c_ = 28.4 mm, *L*_21_ = 79 mm, *L*_m_ = 6.6 mm, *w*_21_ = 33 mm, *s*_w_ = 1.2 mm, *s*_m_ = 0.34 mm, *s*_c21_ = 6.6 mm, and *s*_p_ = 1.2 mm. (**b,c**) the reflection coefficient |*S*_11_| and the insertion loss |*S*_21_| for a range of *d*_p_, respectively. Each cavity has the same four perturbation via-holes. For *d*_p_ in the range of 0.6 mm to 0.8 mm, the lower frequency band at about 5.3 GHz shifts to about 5.36 GHz with the same bandwidth of 90 MHz, but the higher frequency band around 6.32 GHz remains unchanged. For these three cases, the insertion loss is less than 2.3 dB.

**Figure 4:**
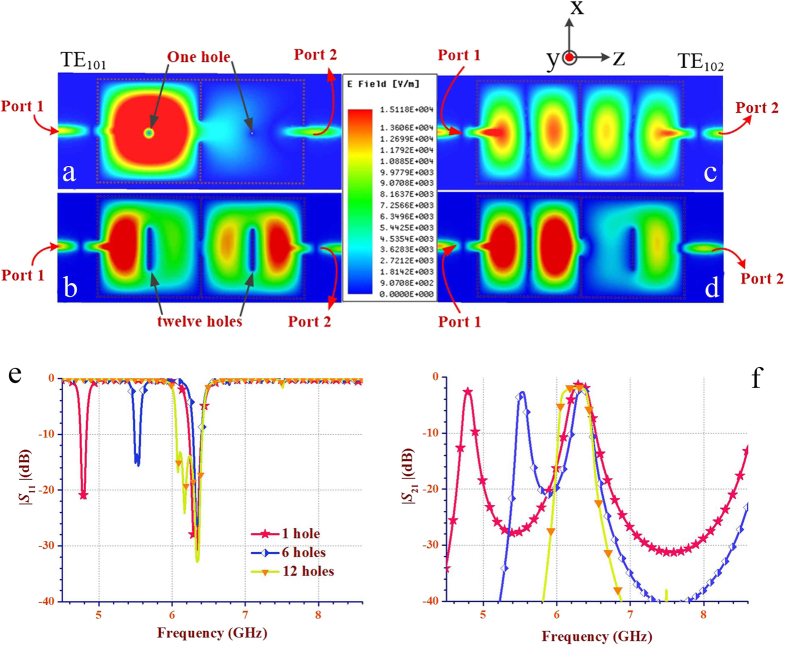
Electric field distributions and simulated scattering-parameters of the two-cavity dual-band SIW filter with different numbers of perturbation via-holes. (**a,b**) Electric field distributions of the TE_101_ mode with 1 and 12 perturbation holes in each cavity, respectively. When the number of perturbation holes varying from 1 to 12, *d*_p_ is fixed as 0.6 mm and the other dimension parameters are the same as in Fig. 3(a). As illustrated in **(a)**, 12 perturbation via-holes have a much greater impact on the electric field distributions of the TE_101_ mode when compared with only 1 perturbation hole. (**c,d**) Electric field distribution of the TE_102_ mode with 1 and 12 perturbation holes in each cavity, respectively. It can be found that different numbers of perturbation via-holes do not have obvious influence on the electric-field distribution profile of the TE_102_ mode. (**e,f**) Curves illustrating the reflection coefficient |*S*_11_| and the insertion loss |*S*_21_| for three different numbers of perturbation via-holes, respectively. As can be seen from (**e**) the lower frequency (the resonant frequency of the TE_101_ mode) band increases from 4.80 GHz to 6.11 GHz with the increasing number of perturbing via-holes, but the higher frequency at 6.33 GHz (the resonant frequency of the TE_102_ mode) is almost unaltered. In particular, when the number of via-holes increases to 12, two different bands generated from both TE_101_ and TE_102_ modes are combined into a single wide band of 6.06 GHz to 6.40 GHz.

Fig. 3(a) illustrates a two-cavity dual-band SIW filter with four perturbation via-holes for sensitivity analysis of the diameter of perturbation via-holes. The coupling coefficient between the two resonators (cavities) can be calculated by^4^:


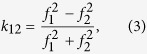
where *f*_1_ and *f*_2_ are the first and second cavity resonant frequencies, respectively. The results depicted in Fig. 3(b,c) indicate that the return loss (namely, reflection coefficient) and the insertion loss of the proposed dual-band dual-mode SIW filters are not sensitive to the diameter of perturbation via-holes. Fig. 4 presents the electric field distributions and the simulated scattering-parameters of the two-cavity dual-band SIW filter with different numbers of perturbation via-holes. As shown in Fig. 4, using different numbers of perturbation via-holes has no obvious effect on the electric- and magnetic-field distribution profiles of the TE_102_ mode, but directly changes the field distribution and the corresponding scattering-parameter performance of the TE_101_ mode. Therefore, the first band can be controlled and tuned by applying different numbers of perturbation via-holes without affecting the second band. Note that when the number of via-holes increases to twelve, two different bands generated from both TE_101_ and TE_102_ modes are combined into a single wide band from 6.06 GHz to 6.40 GHz (i.e., a fractional bandwidth of 5.46%), as depicted in Fig. 4(e,f). This means that the proposed dual-mode SIW filters not only can be advantageous in designing dual-band filters, but also can be effective in the implementation of single-band filters with a relatively wide bandwidth of about 5%.

**Figure 5:**
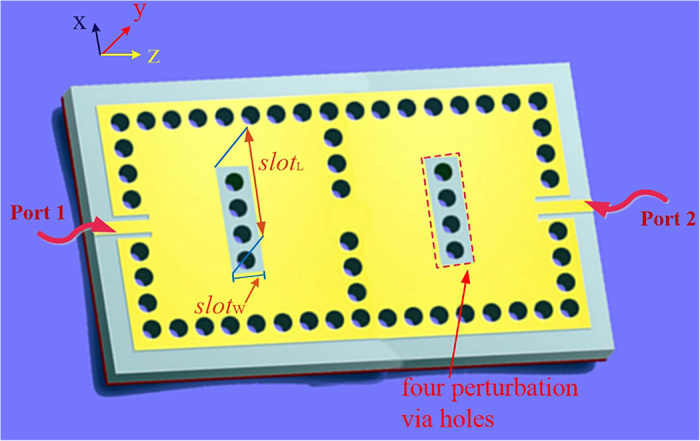
The two-cavity dual-band SIW filter with four perturbation via holes for sensitivity analysis of the slot parameters in each cavity. *L*_21_ = 77 mm, *L*_m_ = 6.9 mm, *L*_c_ = 28.4 mm, *d*_p_ = 0.6 mm, *d*_w_ = 0.6 mm, *w*_21_ = 42 mm, *s*_c21_ = 7.27 mm, *s*_m_ = 0.64 mm, *s*_w_ = 1.2 mm, and *s*_p_ = 1.2 mm. The dimension definitions are the same as in Fig. 3(a). Each added slot of two cavities in this dual-band SIW filter is only on the top metal layer. The length (*slot*_L_) and width (*slot*_w_) of this kind of slot are marked along the *x*-axis and *z*-axis in this figure, respectively.

**Figure 6:**
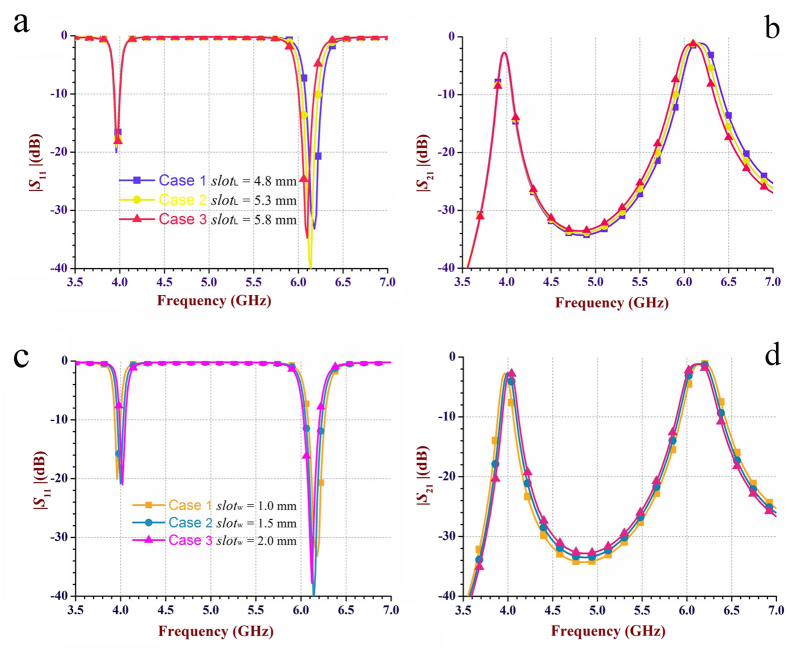
The scattering parameters of the two-cavity dual-band SIW filter with a slot with different lengths and widths in each cavity shown in the Fig. 5. (a,b) The reflection coefficient |*S*_11_| and the insertion loss |*S*_21_| for a range of *slot*_L_ and *slot*_w_ of 1.0 mm, respectively. For *slot*_L_ of 4.8 mm to 5.8 mm, the lower frequency at 3.96 GHz (i.e., the resonant frequency of the TE_101_ mode) is almost unchanged, whereas the higher frequency at 6.2 GHz (i.e., the resonant frequency of the TE_102_ mode) has shifted slightly by less than 80 MHz (i.e., <1.3%). (**c,d**) |*S*_11_| and |*S*_21_| for a range of *slot*_w_ and *slot*_L_ of 4.8 mm, respectively. Note that for *slot*_w_ of 1.0 mm to 2.0 mm, both lower and higher frequencies of 3.96 GHz and 6.2 GHz, respectively are very stable with a slight shift of less than 60 MHz (i.e., <1.5%).

In order to fabricate the reconfigurable dual-band dual-mode SIW filters, adding the required slot around the perturbation via-holes is a popular option from practical implementations point of view. Fig. 5 shows the 3D structure of the proposed two-cavity dual-band SIW filter with the added slot’s parameters. The sensitivity analysis of the slot parameters including the length (*slot*_L_) and width (*slot*_w_) are presented in Fig. 6. For a range of *slot*_L_ and *slot*_w_ as shown in Fig. 6(a–d), respectively, the resonant frequencies for both TE_101_ and TE_102_ modes have shifted marginally by less than 1.5%, which can be neglected or easily corrected in practical circuits and systems.

**Figure 7:**
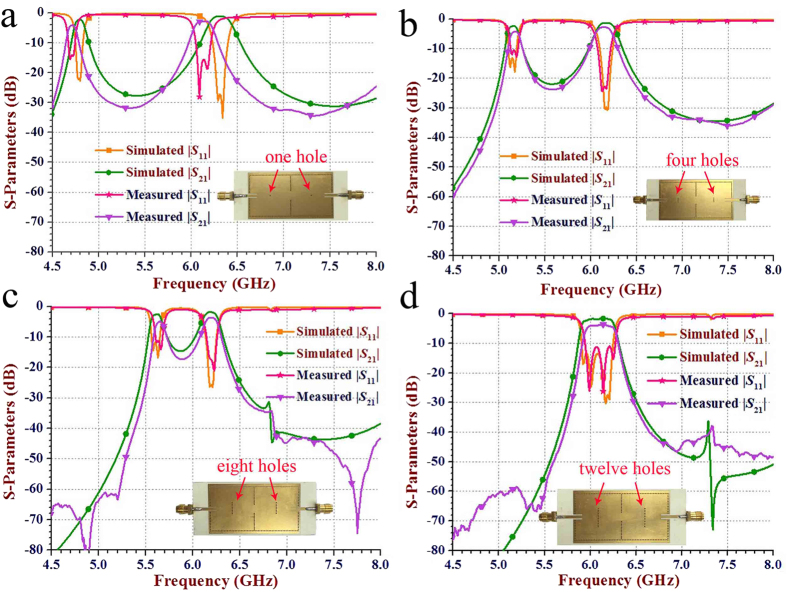
The measured and simulated scattering parameters of the fabricated two-cavity dual-band SIW filters with different numbers (1, 4, 8, and 12) of perturbation via-holes, see as shown in Fig. 3(a). The measured and simulated scattering parameters of the fabricated SIW filter with: (**a**) a single perturbation via-hole and measured lower frequency (*f*_1_) and higher frequencies (*f*_2_) of about 4.71 GHz and 6.14 GHz, respectively, (**b**) 4 perturbation via-holes and measured *f*_1_ and *f*_2_ of about 5.16 GHz and 6.15 GHz, respectively, (**c**) 8 perturbation via-holes and measured *f*_1_ and *f*_2_ of about 5.63 GHz and 6.22 GHz, respectively, and (**d**) 12 perturbation via-holes. The operating band of |*S*_11_| (<−10 dB) is from 5.95 GHz to 6.27 GHz, thus indicating the absolute bandwidth of 320 MHz and the fractional bandwidth of about 5.24%. The images at the right corner in Fig. 7 are the photographs of fabricated dual-band SIW filters, respectively. The circuit dimensions of the proposed four dual-band SIW filters are all 33 × 79 mm^2^.

Subfigures of Fig. 7 depict the measured and simulated scattering parameters of four fabricated two-cavity dual-band SIW filters versus different quantities of perturbation via-holes. In the right corners of the subfigures in Fig. 7, the photographs of the fabricated dual-band SIW filters are exhibited, respectively. The used substrate is Rogers 4350B with a relative permittivity of 3.66. The dimensions of the prototypes of the four dual-band SIW filters are all 33 × 79 mm^2^. As can be seen, there is a good agreement between the simulated and measured results. Considerable deviations are observed in the measured results compared with the simulated ones, which can be attributed to finite conductivity of the metal/via, the fabrication error, the degradation caused by the SMA(SubMiniature version A) connectors. As the number of perturbation via-holes increases from 1 to 8, the measured lower frequency *f*_1_ shifts from 4.71 GHz to 5.63 GHz, thus indicating wide-range frequency tunability for *f*_1_. We have presented the realization of a new wide-band dual-mode SIW filter with 12 perturbation via-holes, as shown in Fig. 7(d) with the measured absolute bandwidth of 320 MHz and the fractional bandwidth of about 5.24%, respectively.

### The reconfigurable dual-band dual-mode SIW filter

**Figure 8:**
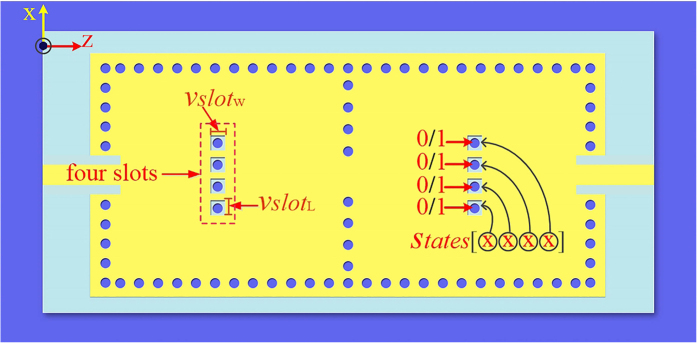
The two-cavity dual-band SIW filter with four reconfigurable perturbation via holes in each cavity. Each perturbation via-hole is surrounded by a rectangular slot of length *vslot*_L_ = 1 mm and width *vslot*_w_ = 1 mm in each cavity. The connections or disconnections between each perturbation hole can readily be controlled with the top metal layer. Note that ‘0’ and ‘1’ refer to ***disconnected*** and ***connected*** perturbation holes, respectively.

**Figure 9:**
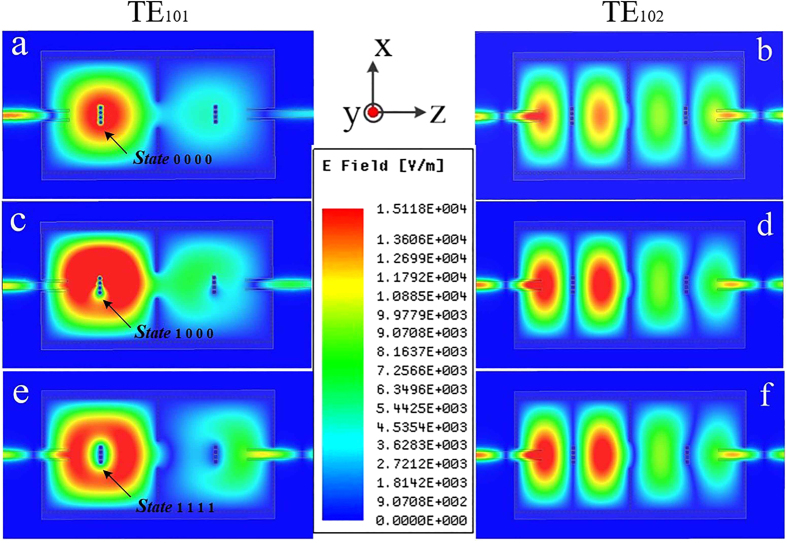
Electric field distributions of the two-cavity dual-band SIW filter in three states. **(a,b**) Electric field distributions of the TE_101_ mode and the TE_102_ mode in *S*tate 0000, respectively. **(c,d**) Electric field distributions of the TE_101_ mode and the TE_102_ mode in *S*tate 1000, respectively. **(e,f**) Electric field distributions of the TE_101_ mode and the TE_102_ mode in *S*tate 1111, respectively. Compare (**a,c,e**) in Fig. 9, and we can find that the perturbation holes connected with the top metal layer disturb the electromagnetic distribution of the TE_101_ mode, while the disconnected ones will not affect the electromagnetic distribution of the TE_101_ mode. Furthermore, both the perturbation holes disconnected and connected cannot affect the electromagnetic distribution of the TE_102_ mode as shown in (**b,d,f**).

**Figure 10:**
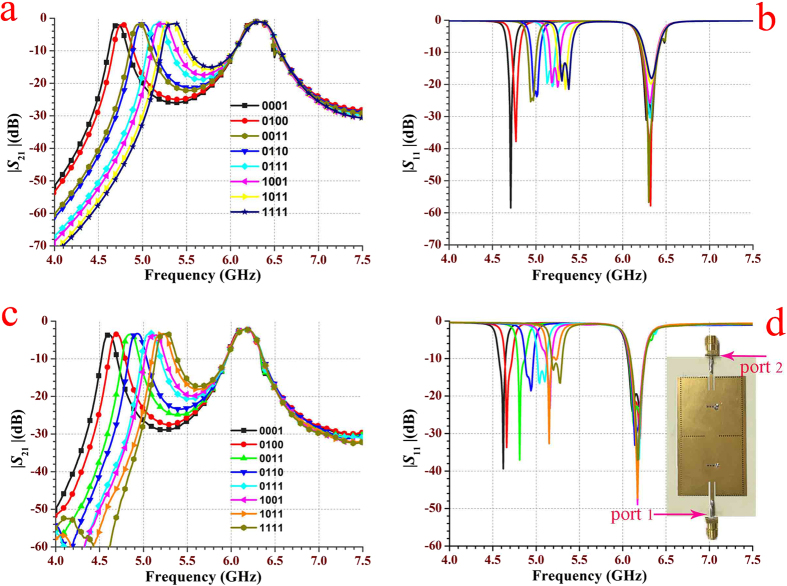
The simulated and measured 8-state scattering parameters of the fabricated two-cavity dual-band SIW filter shown in Fig. 8 with four reconfigurable perturbation via-holes in each cavity. (a,b) The simulated scattering parameters. (**c,d**) The measured scattering parameters. The measured results show that when the state varies from [0001] to [1111], the lower frequency (the resonant frequency of the TE_101_ mode) is tuned from 4.61 GHz to 5.24 GHz, whereas the higher frequency (the resonant frequency of the TE_102_ mode) is almost unchanged at around 6.18 GHz. Note that the subfigure at the right corner in the (**d**) is the photograph of this fabricated reconfigurable dual-band SIW filter. The circuit dimension of the proposed reconfigurable dual-band SIW filter is 42 × 79 mm^2^.

Fig. 8 illustrates the two-cavity dual-band SIW filter with 4 reconfigurable perturbation via-holes per cavity. The used substrate is Rogers 4350B, which is consistent with the used substrate in the previous section. However, its relative permittivity is 3.48, because of the different batches of the substrate. Different to the perturbation via-holes in Fig. 3(a), each one in Fig. 8 is surrounded by a rectangular slot. Slots are designed to facilitate the control of the connection states between perturbation holes and the top metalized layer. In this paper, the control of connection states in measurement is simply achieved through soldering. Nevertheless, in practical production, the states can be controlled by electrical switches, mechanical switches, nanomaterial switches, *etc*. Due to the reconfigurable feature of 4 perturbation via-holes with different states, the fabricated SIW filter has 16 states to control the final dual-band performance. Fig. 9 depicts electric field distributions of the two-cavity dual-band SIW filter in three typical states, *S*tate 0000, *S*tate 1000 and *S*tate 1111. As shown in Fig. 9(a,c,e), the perturbation holes connected to the top metalized layer perturb the electromagnetic distribution of the TE_101_ mode, while the disconnected ones do not affect the electromagnetic distribution of this mode. Fig. 9(b,d,f) indicates that both the disconnected and connected perturbation holes cannot affect the electromagnetic distribution of the TE_102_ mode. Fig. 10 illustrates the simulated and measured 8-state scattering parameters of the fabricated two-cavity dual-band SIW filter with 4 reconfigurable perturbation via-holes per cavity. With the state varying from [0001] to [1111], the measured lower frequency (the resonant frequency of the TE_101_ mode) is tuned from 4.61 GHz to 5.24 GHz, whereas the measured higher frequency (the resonant frequency of the TE_102_ mode) is unchanged at about 6.18 GHz. The simulated and measured curves are consistent with the explanation made in Fig. 9. The image at the right corner in Fig. 10(d) is the photograph of this fabricated reconfigurable dual-band SIW filter. The circuit dimension of the proposed reconfigurable dual-band SIW filter is 42 × 79 mm^2^. These results confirm and verify the operation of a novel dual-band dual-mode SIW filter with the independently reconfigurable TE_101_ resonant mode.

## Discussion

This paper presents a novel realization of dual-band or wide-band dual-mode substrate integrated waveguide (SIW) filters based on additional metalized via-holes. The main feature of the proposed approach is that the independent perturbation can be introduced to the first resonant mode TE_101_, but the second resonant mode TE_102_ is not affected. Based on the analysis of the operating principle and sensitivity studies of parameters, four two-cavity dual-band SIW filters and a reconfigurable two-cavity dual-band SIW filter are designed, simulated, fabricated and theoretically and experimentally verified. The measured results indicate that the available measured frequency-ratio ranging from 1 (wide-band case) to 1.3 (dual narrow band case with the largest separation) can be realized in four two-cavity dual-band SIW filters, and the center frequency of the first band can be tuned from 4.61 GHz to 5.24 GHz but the center frequency of the second one will be unaltered and fixed at near 6.18 GHz in the two-cavity dual-band SIW filter with four reconfigurable via-holes. In summary, the proposed perturbation approach by using the additional metalized via-holes in dual-band or wide-band even tunable band SIW filters offers several advantages including 1) high selectivity of dual band or wide band, 2) low-cost fabrication using printed circuit board technology, 3) easy integration with single-layer circuits and systems, 4) convenient implementation of tunable or reconfigurable band, and 5) enhanced power capability and decreased radiation loss due to complete shielding with standard metalized via-holes. The fixed or tunable dual-band filters can be used to construct a multi-mode and multi-band filter array. In addition, it can be used in a Cognitive Radio System, an FM Communication System and a Radar RF System. This paper provides a method for designing a dual-band SIW filter with a tunable lower passband and a fixed upper passband, and the frequency ratio of dual-band can be adjusted regularly. It can be applied in the particular dual-band transceiver system that the higher passband needs to be fixed while the lower passband can be switched flexibly. Nevertheless, for a system requiring arbitrary dual-band frequencies, the fixed band can be obtained by altering the overall dimensions of this SIW filter, while the other band can be obtained by utilizing the method proposed in this paper. Hence, the proposed method is universal for certain system frequencies. In conclusion, this work provides not only a solution for designing a dual-band SIW filter, but also universal strategies and thoughts for realizing reconfigurable filters.

## Additional Information

**How to cite this article**: Wu, Y. *et al*. Dual-Band Dual-Mode Substrate Integrated Waveguide Filters with Independently Reconfigurable TE_101_ Resonant Mode. *Sci. Rep.* **6**, 31922; doi: 10.1038/srep31922 (2016).
